# Surface Hydrophilicity and Antifungal Properties of TiO_2_ Films Coated on a Co-Cr Substrate

**DOI:** 10.1155/2017/2054723

**Published:** 2017-08-07

**Authors:** Lijuan Huang, Shuanglin Jing, Ou Zhuo, Xiangfeng Meng, Xizhang Wang

**Affiliations:** ^1^Department of Prosthodontics, Nanjing Stomatological Hospital, Medical School of Nanjing University, 30 Zhongyang Road, Nanjing, Jiangsu 210008, China; ^2^Jiangsu Key Laboratory of Oral Diseases and Department of Endodontics, Affiliated Hospital of Stomatology, Nanjing Medical University, 136 Hanzhong Road, Nanjing, Jiangsu 210029, China; ^3^Key Laboratory of Mesoscopic Chemistry of MOE, School of Chemistry and Chemical Engineering, Nanjing University, Nanjing 210093, China

## Abstract

The purpose of this study was to deposit a thin layer of TiO_2_ on a Co-Cr substrate, serving as a deactivation film protecting the metallic fitting surface. The crystalline structure and surface morphology of the film were characterized by X-ray diffraction (XRD) and scanning electron microscopy (SEM). A scratch tester was used to examine the adhesion strength between the TiO_2_ film and the Co-Cr substrate. The water contact angles and antifungal efficacy against* C. albicans* of the TiO_2_-deposited Co-Cr samples were investigated and further compared with those of uncoated Co-Cr substrates. The results indicated that a pure anatase microstructure and dense and smooth surface texture as well as strong binding to the underlying metallic surface were obtained. The originally hydrophobic Co-Cr alloy surface turned hydrophilic after TiO_2_ film coating. Most importantly, the TiO_2_-coated surface showed a superior antifungal capability under UV-irradiation compared to those without TiO_2_ coating. This work contains meaningful results for the development of a new metallic framework coating with improved hydrophilicity and antifungal properties.

## 1. Introduction

The demand for prosthetic treatment is of increasing tendency as a result of the aging issue globally [1, 2]. Among all available techniques, removable partial denture (RPD) is regarded as a simple and economic option to repair the missing teeth [3]. Therefore, RPDs have been the commonly used treatment for edentulism to date. Co-Cr alloys have a wide range of applications as a material for metallic framework owing to their good mechanical properties as well as superior biocompatibility. However, it has been reported that yeasts and bacteria are prone to colonize on the fitting surface of the dentures forming biofilms [4, 5]. These biofilms consequently cause damage to the oral mucosa below, commonly associated with denture stomatitis (DS). DS affects up to two-thirds or even more of denture wearers, the majority of whom are asymptomatic and unaware of the problem [6–8].

The etiology of DS is multifactorial, among which the* candida* primarily* C. albicans* infection and poor denture hygiene have been widely accepted as critical risk factors [9–11]. Yoshijima et al. [12] have reported an association of hydrophobic surface with the colonization of* C. albicans*. It is therefore believed that a proper deactivation of the metallic fitting surface may alleviate the problem associated with biofilms. This is attributed to the increase of hydrophilicity and the antimicrobial properties relying on surface coating techniques. Recently, titanium dioxide (TiO_2_) coating has drawn great attentions due to its photocatalytic, antimicrobial, and self-cleaning properties [13–17]. In addition, TiO_2_ films have shown enhanced biocompatibilities and good corrosion resistance of the substrate [18, 19]. In the published studies, TiO_2_ thin film has been applied to denture base acrylic resin, resulting in increased hydrophilic properties and decreased attachment of food bolus accumulation, as well as an inhibitory effect on adhesion of microorganisms [20, 21]. However, to the best of our knowledge, the study of TiO_2_ film on Co-Cr alloy frameworks is still not available, since TiO_2_ films growing on a Co-Cr alloy surface with good bonding strength are challenging, restricting their applications so far.

A large magnitude of techniques, including sol-gel processes, chemical vapor deposition, electrophoretic deposition, plasma immersion ion implantation, plasma spray, and atomic layer deposition (ALD), have been made available for depositing TiO_2_ films on a variety of surfaces [22–25]. Among the aforementioned techniques, ALD has been widely applied due to its figure of merits including high-quality and large-area flat coating, perfect structure, and process controllability. Although significant efforts have been devoted to depositing TiO_2_ films on various substrates, such as silicon and metallic and polymer materials [23, 26, 27], there is little information in the literature with regard to the application of ALD in dentistry. Therefore, this study was innovatively designed to deposit a thin layer of TiO_2_ on a Co-Cr substrate, with the help of ALD. Concerning potential dental applications, our hypothesis was that the hydrophilic and antimicrobial capacity of TiO_2_ films might have the potential to inhibit the growth of* C. albicans *adhered to the metallic frameworks and thus lower the risk of associated denture stomatitis. In particular, an optimized ALD process was adopted via a home-made ALD system. The crystalline structure, microstructure, and adhesion strength of deposited TiO_2_ film were examined. Moreover, the water contact angles and antifungal properties of the TiO_2_-deposited Co-Cr samples were investigated and further compared with those of uncoated Co-Cr substrates.

## 2. Materials and Methods

### 2.1. Preparation of TiO_2_ Film

The experiments were carried out in a hot-wall ALD system (home-made system, School of Chemistry and Chemical Engineering, Nanjing University, China). The Co-Cr alloy (Bego, Bremen, Germany) was used as substrate material, with the alloy composition given in Table 1. Small disc samples (diameter = 15 mm; thickness = 2.5 mm) were casted from the Co-Cr alloy. Prior to deposition, the substrate specimens were mechanically grounded by water-proof SiC abrasive papers with various sizes of up to 2000 grit and then polished with 30–50 nm alumina powder. The alloy discs were further ultrasonically cleaned in diluted sodium hydroxide solution, acetone, ethanol, and deionized water sequentially to remove residual surface contamination completely and finally dried N_2_.

**Figure 1:**
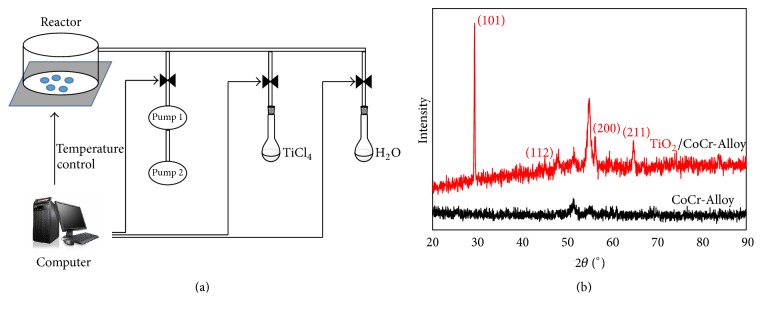
(a) Sketch of ALD technique (Pump 1: turbomolecular pump. Pump 2: sliding-vane rotary vacuum pump) and (b) XRD pattern of the surface of the treated sample.

TiCl_4_ and water, held in separate external reservoirs at room temperature, were alternatively introduced into the reactor chamber. The sketch is shown in Figure 1(a). One ALD deposition cycle consists of 3.0 s TiCl_4_ pulse time, 250 s pump-down, 2.0 s water pulse time, and 300 s pump-down, respectively. Herein, a longer pump-down period was employed instead of using high purity N_2_/Ar gas as purging gas to remove any residual reactants and by-products. The deposition temperature was held at 300°C constantly and 1000 ALD cycles were deposited for each sample.

### 2.2. Film Characterization Measurements

The crystalline structure of films was identified by X-ray diffraction (XRD) (D8, Bruker AXS GmbH, Karlsruhe, Germany). CoK*α*_1_ radiation (*λ* = 0.178897 nm) was over a range from 20° to 90° (2*θ*). The surface morphology of the samples was examined by scanning electron microscopy (SEM) (S-3400N II, Hitachi, Tokyo, Japan). The cross-sectional microstructure and thickness of deposited films were determined by field emission scanning electron microscopy (Quanta FEG 250, FEI, Hillsboro, America).

A scratch tester was used to examine the adhesion strength between the TiO_2_ film and the Co-Cr substrate (UMT Multi-Specimen Test System, CETR, CA, USA). The coated surface was scratched using a conical Rockwell C tip with diameter of 5 *μ*m. Scratch (3 mm in length) was made with the applied loads ranged from 0.15 to 5.0 Kg at a linear speed of 0.01 mm/s, where the acoustic emission (AE) signal intensity (due to interface exfoliating) was monitored at the same time. The critical load was identified with the continuous increase in the AE signal during scratching. After testing, the scratch on the surface of TiO_2_ film was evaluated by optical microscope (Gx41, Olympus, Tokyo, Japan) at 16x magnification.

The static contact angles of coated and uncoated samples were measured using the sessile drop method at room temperature (OCA30 video contact angle system, Dataphysics, Filderstadt, Germany). A 2.0 *μ*L droplet of deionized water was dropped on the testing surface using a computer-controlled microsyringe. The contact angle was then determined from the magnified image collected with a camera in the system. Six samples from each group were selected randomly and three replicate measurements were performed at different positions. The averaged value was presented as the contact angle for each sample. The data is presented as the mean ± standard deviation. A *t* test was applied for statistical analysis, with *P* < 0.05 being considered significant.

### 2.3. Antifungal Property Tests

*C. albicans* strain (ATCC 10231) was used as the pathogen for the microbiological tests. Cultures of microorganisms were grown in Martin Broth, modified (liquid) at 37°C with 150 rpm shaking for 12 h. 10-fold serial dilutions of the incubation fluid were applied to reach the concentration of 10^7^ colony-forming units per milliliter (CFU/ml). The tested samples were sterilized under ultraviolet light (245 nm) for 2 h, prior to the investigations. A total of 100 *μ*L* C. albicans* diluted suspension was added onto the TiO_2_-coated and uncoated surface, respectively. After illumination under an 8 W UV lamp (365 nm) for 1 h, the droplets were washed from the sample surface using 0.9 ml saline solution repeatedly. Afterwards 100 *μ*L of each serially diluted washing suspension was dispersed on the modified Martin Agar Medium and incubated for 24 h at 37°C. The number of colonies on the mediums was quantified via the direct counting method after incubation.

## 3. Results

### 3.1. Structural Characterization of TiO_2_ Film

Figure 1(b) displayed a typical XRD spectrum of the TiO_2_-deposited Co-Cr sample. Besides the typical peaks corresponding to elements of Co-Cr substrate, the dominating (101), (112), (200), and (211) crystalline peaks corresponded to the anatase TiO_2_ phase. This suggested a highly crystalline TiO_2_ film formed on the Co-Cr alloy. Anatase TiO_2_ was the dominating crystalline form of the surface layer under current experimental conditions.

**Figure 2:**
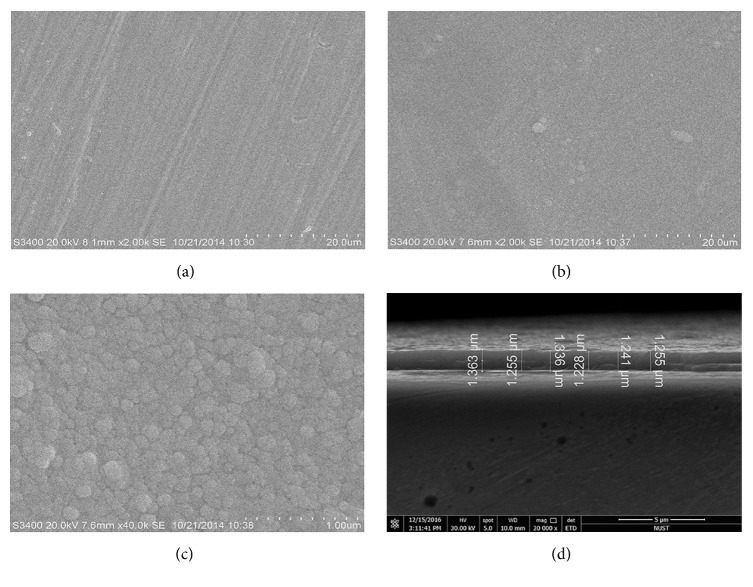
SEM images of (a) Co-Cr substrate in low magnification. (b) TiO_2_ film in low magnification. (c) TiO_2_ film in high magnification. (d) Cross-sectional view of TiO_2_ film on Co-Cr substrate.

The microstructures of the sample with and without TiO_2_ film were shown in SEM images (Figures 2(a)–2(c)). The surface of the TiO_2_ film was relatively dense, uniform, and smooth without noticeable pinholes or cracks (Figure 2(b)). It was noted that the TiO_2_ film has entirely covered the polishing scratches on the Co-Cr surface (Figure 2(a)). In addition, a large number of rounded grains with the size of about 100 nm were observed in a high-magnification SEM image of TiO_2_ film (Figure 2(c)). It could be seen from the cross-sectional scanning of the TiO_2_-coated sample that the TiO_2_ layer was continuous, and its thickness was estimated to be 1.2–1.4 *μ*m (Figure 2(d)).

### 3.2. Adhesion Strength

**Figure 3:**
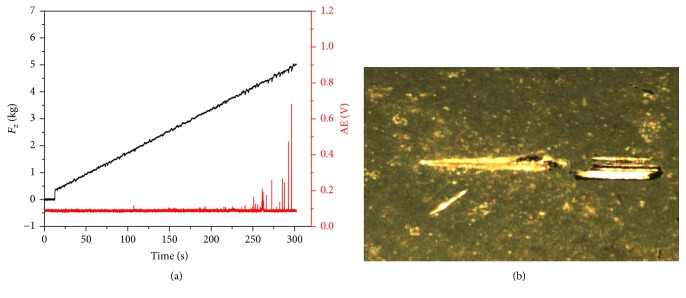
Scratch test (a) applied load and AE signal intensity. (b) Optical image of the scratch test specimen.

The adhesion strength between the coating layer and substrate is listed as one of the most important factors determining the lifetime and performance of the coated component. The association curve between AE signal and the load on C tip showed that the TiO_2_ coating starts to peel off when the load reached approximately 4.0 Kg with continuously peaks (Figure 3(a)). The scratch morphology image (Figure 3(b)) revealed neither observed neighboring cracks nor coating detachment, besides the scratch corresponding to the applied force. All aforementioned observations suggested a good adhesion between the TiO_2_ film and Co-Cr substrate.

### 3.3. Water Contact Angle

**Figure 4:**
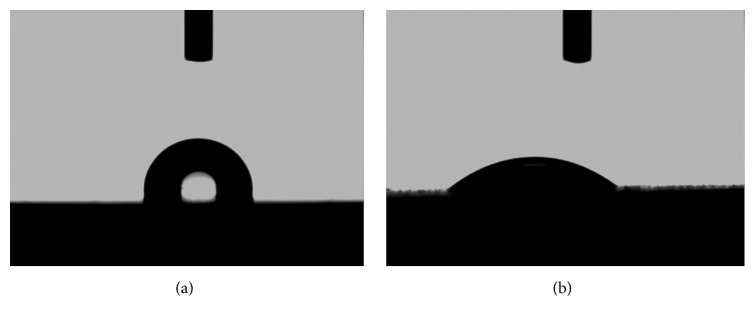
Optical images of a water droplet in contact with Co-Cr (a) and TiO_2_ (b) surface.

The optical images of water drops sprayed onto the tested samples demonstrated that the average surface contact angle of deionized water was 103.0° ± 1.2° on the Co-Cr alloy and 37.3° ± 3.8° on the TiO_2_ -coated samples, respectively (Figure 4), indicating that the water contact angle of Co-Cr alloy showed a significant decrease (*P* < 0.05) after the TiO_2_ film deposition.

### 3.4. TiO_2_-Coated Film Exhibited a Powerful Antifungal Property

**Figure 5:**
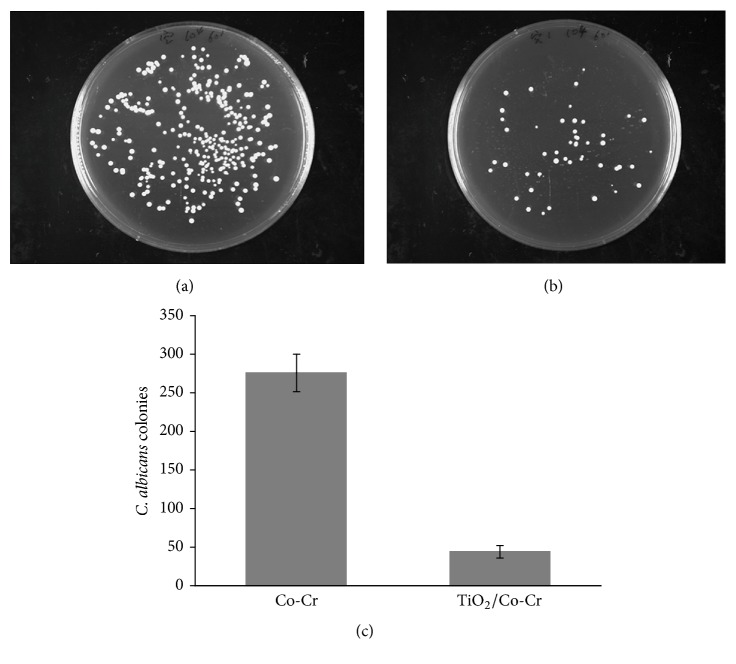
Photo images of incubated* C. albicans* colonies washed from surface of uncoated (a) and TiO_2_ -coated sample (b). (c) The numbers of* C. albicans* colonies.

In order to clarify the relative number of survival* C. albicans* on tested surface after 1 h UV illumination, the diluted suspensions washed from the uncoated and TiO_2_-coated sample surface were incubated, respectively. Figures 5(a) and 5(b) showed photo images of colonies on the media after incubation for 24 h. The number of survival* C. albicans* on TiO_2_-coated sample was much lesser than that on uncoated one. As shown in Figure 5(c), the quantified number of colonies from TiO_2_-coated group was significantly less than uncoated group (*P* < 0.05). These results demonstrated that the TiO_2_ film presented a powerful antifungal effect under UV illumination.

## 4. Discussion

The majority of ALD reactions rely on two gaseous precursors introduced into the chamber in a sequential, self-limiting manner. An inert gas, such as Ar and nitrogen, is infused between the precursor pulses to remove extra reactants and by-products. For example, Cheng et al. obtained TiO_2_ films using TiCl_4_ and H_2_O, employing Ar as the purging gas, on a magnitude of substrate materials [28]. In the current study, the application of ALD has been successfully extended to coat TiO_2_ films on a Co-Cr alloy. Moreover, the method optimization includes longer pump-down instead of purging gas. This modification greatly simplified the experimental procedure by obviating additional purging gases. On the other hand, the experimental period was slightly extended. Further optimization in terms of pumping capacity and tubing design will help to improve the working efficiency of current setup.

It is well-known that the phase composition and photocatalytic properties of the ALD TiO_2_ films are dependent on many parameters such as the chamber pressure, the deposition temperature, and the substrate material. On the basis of XRD pattern, it was concluded that the film grown under the current experimental conditions at 300°C was pure anatase structure. Earlier studies have also shown that the anatase structure with lower conduction* bandedge* exhibits a stronger photocatalytic and bactericidal ability [22, 28–30].

The surface smoothness is considered as an important factor influencing the adherence of oral pathogenic microorganisms [31–33]. Surface scratches and cracks can enhance the attachment of microorganisms and the growth of biofilms [8, 34]. Furthermore, wettability is also an important property of biomaterials because hydrophilic surfaces are more resistant to microbial adhesion than hydrophobic surfaces [12, 35, 36]. Usually, surface with a contact angle more than 70° is identified as hydrophobic, while a hydrophilic surface has a contact angle below 70° [37]. Results from the water contact angle measurements suggested that the Co-Cr alloy was hydrophobic and then became hydrophilic when coated with the TiO_2_ film. Yoshijima et al. [12] have reported that the decreasing surface hydrophobicity can diminish the ability of* C. albicans* to attach and colonize the denture surface. Our results revealed that coating TiO_2_ film can cover the polished scratches and increase the surface hydrophilicity of Co-Cr alloy at the same time. This may prevent the microbial attachment to certain extent and thus resist the development of biofilms leading to denture stomatitis.

More importantly, some studies have identified TiO_2_ as a surface coating to enhance the antibacterial capability of substrate materials [13, 26, 27]. This observation is most likely due to its photo-induced superhydrophilicity and photocatalytic property, though the exact bactericidal mechanisms of TiO_2_ under UV-irradiation are still under debate [22]. In general, the cell wall and membrane damage by reactive oxygen species generated from the photocatalytic activity of TiO_2_ is the mostly accepted killing action [38]. Furthermore, not only bacteria but also viruses, fungi, and other microorganisms can also be killed by UV-irradiated TiO_2_ [39]. Evaluation of microbial survivability is one of* in vitro* techniques to assess antimicrobial properties [22]. Our study complemented the previous studies by evaluating growth inhibition of* C. albicans* on TiO_2_-coated and uncoated samples. As can be observed from the antifungal experiment, the TiO_2_ coating exhibited a great inhibitory effect against* C. albicans*, with a statistically significant reduction in the number of colony-forming units (CFU) compared to the control group (*P* < 0.05). Thus, it is fair to assume that the powerful oxidative ability of TiO_2_ can induce the apoptosis and necrosis in* C. albicans* cells, although the multilayer composition of* C. albicans* cell wall is more complicated and resistant to antimicrobial activity than that of bacteria [40]. To better understand the underlying mechanism and mimic the real scenario, it is beneficial to perform a time-resolved antifungal experiment lasting a longer period of days or even weeks. Moreover, the illuminating UV conditions in terms of strength and duration can be further studied to match more practical values.

## 5. Conclusion

In conclusion, TiO_2_ thin coatings have been successfully deposited on Co-Cr substrate via an optimized ALD process. The figure of merits included pure anatase structure, dense and smooth surface, strong bonding, and full coverage to the substrate surface. More importantly, the TiO_2_ coatings possessed a high antifungal activity that eliminates most of the* C. albicans* after the UV-irradiation. Therefore, there is a great potential of TiO_2_ coating on future RPD items against possible denture stomatitis.

## Figures and Tables

**Table 1:** Chemical composition of the Co-Cr alloy.

Element	Co	Cr	Mo	Ni	Others
(wt.%)	62.3%	29.3%	6.2%	1.0%	1.2%
